# Functional Correlates of Resting-State Connectivity in the Default Mode Network of Heroin Users on Methadone Treatment and Medication-Free Therapeutic Community Program

**DOI:** 10.3389/fpsyt.2019.00381

**Published:** 2019-06-06

**Authors:** Li-Wei Kuo, Pei-Sheng Lin, Shih-Yen Lin, Ming-Fang Liu, Hengtai Jan, Hsin-Chien Lee, Sheng-Chang Wang

**Affiliations:** ^1^Institute of Biomedical Engineering and Nanomedicine, National Health Research Institutes (NHRI), Miaoli, Taiwan; ^2^Institute of Medical Device and Imaging, National Taiwan University College of Medicine, Taipei, Taiwan; ^3^Institute of Population Health Sciences, NHRI, Miaoli, Taiwan; ^4^Department of Computer Science, National Chiao Tung University, Hsinchu, Taiwan; ^5^Research Center of Sleep Medicine, College of Medicine, Taipei Medical University, Taipei, Taiwan; ^6^Department of Psychiatry, School of Medicine, College of Medicine, Taipei Medical University, Taipei, Taiwan; ^7^Department of Psychiatry, Shuang-Ho Hospital, Taipei Medical University, New Taipei City, Taiwan; ^8^Center for Neuropsychiatric Research, NHRI, Miaoli, Taiwan; ^9^Department of Psychiatry, Cardinal Tien Hospital, New Taipei City, Taiwan

**Keywords:** heroin, methadone, therapeutic community, resting-state functional magnetic resonance imaging, Cambridge gambling task, default mode network

## Abstract

The treatment of heroin addiction is a complex process involving changes in addictive behavior and brain functioning. The goal of this study was to explore the brain default mode network (DMN) functional connectivity using resting-state functional magnetic resonance imaging (rs-fMRI) and decision-making performance based on the Cambridge gambling task in heroin-dependent individuals undergoing methadone treatment (MT, *n* = 11) and medication-free faith-based therapeutic community program (TC, *n* = 11). The DMN involved the medial prefrontal cortex (mPFC), left inferior parietal lobe (IPL_L_), right inferior parietal lobe (IPL_R_), and posterior cingulate cortex (PCC) subregions for all participants in both the MT and TC groups. Compared with MT, TC had an increased functional connectivity in IPL_L_–IPL_R_ and IPL_R_–PCC and decreased functional connectivity in mPFC–IPL_L_ and IPL_L_–PCC. Both groups exhibited no significant difference in the regional rs-fMRI metric [i.e., amplitude of low-frequency fluctuation (ALFF)]. In the analysis of the neural correlates for decision-making performance, risk adjustment was positively associated with ALFF in IPL_L_ for all participants considering the group effects. The involvement of IPL in decision-making performance and treatment response among heroin-dependent patients warrants further investigation.

## Introduction

Heroin addiction is a chronic brain disorder involving interacting neural systems that lead to complex addictive behaviors (1). Medication-assisted treatment (MAT) has played a major role in reducing the harmful consequences related to heroin misuse, with methadone being the most widely used opioid-substitutive agent (2). In addition to the pharmacological intervention, a safe and supportive environment is essential for recovery from addiction. Residential treatment models, such as therapeutic community (TC), have been developed to restructure the thinking, lifestyle, and interpersonal relationships of individuals with opiate dependence. A large body of evidence has demonstrated the effectiveness of MAT and residential treatment programs; however, for heroin users, to be engaged in treatment and continue to be drug-free remain a challenge.

Impaired decision-making, manifested by compulsive drug-taking without consideration of the harmful consequences, is a core feature of drug abusers. The Iowa gambling task (IGT), a paradigm requiring participants to choose between “risky” options with larger short-term gains offset by greater long-term losses and “safe” ones with smaller short-term gains and losses from an ambiguous and learning context, has been extensively used to measure decision-making performance (3). It revealed that heroin-dependent individuals had disadvantageous performance with preference of immediate monetary gain regardless of the long-term loss in IGT (4–6). Stress, duration of abstinence, exposure to drug-related cues, psychopathic traits, and childhood attentional deficit might determine the IGT performance in this population (6–9). The Cambridge gambling task (CGT) has been developed to assess decision-making and risk-taking behaviors outside a learning context. In the CGT, participants receive the relevant information explicitly, and they are required to make a probabilistic decision for each independent trial, then gamble on their confidence in this decision (10). Recently, active heroin users, methadone-treated individuals, and abstinent heroin users were reported to have impaired risk adjustment (RA) with deficit to evaluate the risk probability and calibrate their betting behavior in CGT (11).

The recent advance in resting-state functional magnetic resonance imaging (rs-fMRI) has shed light on the underpinnings of neurocognitive and neurobiological mechanisms in drug addiction and its treatment through the analysis of the circuit-level interactions between brain regions. A previous study investigated the association of rs-fMRI metric, amplitude low-frequency fluctuation (ALFF), and methadone dose among heroin users and normal controls (12). They discovered that heroin users had decreased ALFFs in several brain regions, including the bilateral dorsal anterior cingulate cortex, bilateral medial orbit frontal cortex, left dorsal lateral prefrontal cortex, left middle temporal gyrus, left inferior temporal gyrus, posterior cingulate cortex (PCC), and left cuneus. In addition, increased ALFFs in the bilateral angular gyrus, bilateral precuneus, bilateral supramarginal gyrus, left post cingulate cortex, and left middle frontal gyrus were noted in heroin users. Interestingly, they also reported that the ALFFs in the bilateral parietal lobe were positively correlated with the methadone dose. Another study employing computational classification revealed high accuracy in characterizing the discrepancy of rs-fMRI patterns between heroin-dependent individuals and healthy controls (13). The aforementioned studies demonstrate the capability of the rs-fMRI technique to investigate the brain activities of heroin users.

Previous studies have also suggested that the rs-fMRI functional connectivity in default mode network (DMN) might be altered among heroin users. Li et al. reported that heroin users had a disrupted functional connectivity mainly in the medial prefrontal cortex (mPFC) but not in other DMN subregions (14). However, Ma et al. discovered a decreased rs-fMRI functional connectivity between the PCC/precuneus and bilateral parahippocampal gyrus and medial prefrontal cortex (mPFC) (15). An earlier study also revealed an increased functional connectivity in the right hippocampus and decreased functional connectivity in the right dorsal anterior cingulate cortex and left caudate among heroin users (16).

Moreover, heroin users’ outcome status might be associated with DMN functioning. Relapsed heroin users were reported to have their DMN functional connectivity decreased in the left inferior temporal gyrus and right superior occipital gyrus but increased in the left precuneus and right middle cingulum, compared with abstinent individuals undergoing a methadone treatment (MT) (17). Most existing studies have addressed the relationship between heroin use and brain functioning by comparing healthy controls and short-term abstinent individuals. However, little is known about the interplay between brain functioning and neurocognitive performance for heroin users undergoing treatment and in a recovery process. Therefore, the objective of the present study was to explore and compare the functional connectivity of DMN and the decision-making performance according to CGT in heroin-dependent individuals undergoing MT and TC programs. The first group was continuously exposed to methadone with or without heroin; however, the second group was free from opioid drugs, including heroin and methadone, and other psychoactive agents.

## Materials and Methods

### Participants Recruitment

Two groups of heroin-dependent individuals were enrolled, one in MT and the other undergoing a medication-free TC program. The participants in MT were recruited from the Far Eastern Memorial Hospital (New Taipei City, Taiwan) and En Chu Kong Hospital (New Taipei City, Taiwan); both hospitals provided low-threshold MT programs. The participants in TC programs were recruited from Operation Dawn, a Christian-based organization providing an 18-month residential program for individuals with drug use problems. An advanced 3-year voluntary missionary training program was provided for those who completed the basic programs. The TC programs were abstinence-oriented, and the recovering drug users and professionals provided supportive care and spiritual guidance for drug users to reconstruct their orderly lives and reshape their values. Psychoactive substances, including coffee and tobacco, were prohibited. Psychopharmacological medications were also not allowed; therefore, individuals with disturbing mental symptoms requiring pharmacological treatment were excluded from TC programs.

The enrolled MT and TC participants had to meet the following criteria: 1) aged 20 or above, 2) a lifetime diagnosis of opioid dependence, on the basis of the SCID-I (Structured Clinical Interview for Diagnostic and Statistical Manual of Mental Disorders, Fourth Edition, Text Revision (DSM-IV-TR), Axis-I disorder), and 3) remaining in treatment (MT or TC) for at least 3 months. Given that the participants in TC programs were separated by gender, this study only enrolled male participants based on the accessibility. The study was approved by the institutional review board of the National Health Research Institutes (Miaoli, Taiwan). A total of 11 MT and 11 TC individuals were enrolled between August 2010 and July 2011. Written informed consent was obtained from all participants.

### Clinical Assessments

All participants were interviewed by research nurses. Sociodemographics (age, education, religion) and clinical information (onset of heroin use, current treatment duration, current daily dose of methadone for MT) were collected. The SCID-I was used to assess the current comorbid mood, psychotic, and anxiety disorders (18). The severity of drug use, indexed by the days of drug use in the past 4 weeks, was assessed using the Treatment Outcome Profiles (19). Handedness was assessed using the Briggs–Nebes modified Annett’s 12-item Hand Preference Questionnaire with scores ranging from −100 for extreme left hand preference to +100 for extreme right hand preference (20). Urine drug testing for morphine and amphetamine was performed before MR experiments.

The decision-making capability was assessed using a computerized CGT, a subset of the Cambridge Neuropsychological Test Automated Battery (Cambridge Cognition Ltd). The task was composed of eight blocks of nine trials. At the beginning of each block, a total of 100 cumulative points were offered to participants. On each trial, the participants were presented with an array of 10 blue and red boxes. The ratio of box colors varied pseudo-randomly over the course of the task. Participants were informed that a token had been hidden inside one of these boxes, and they had to decide whether the token had been hidden inside a red or blue box. After choosing the box color (blue or red), participants were required to bet on their decision. Five available bet options, which represented 5%, 25%, 50%, 75%, and 95% of the cumulative points, were offered in an ascending or descending sequence. In the ascend condition (half the blocks), the available bet was 5% upwards, and vice versa for the descend condition (half the blocks). In betting, participants had to decide and wait for their desirable choice (21). Several key features of CGT should be highlighted. First, the varied ratio of box colors provides the explicit outcome probability of gain and loss, which enables the assessment of the decision-making performance under risk. Second, the variable proportion of cumulative points of the bets reflects the willingness to risk on the already acquired wage for further unknown reward. Finally, the inclusion of both ascend and descend conditions allows for distinguishing the participants’ impulsive and risk-taking betting strategies. Risk adjustment (RA) is defined by the degree to which participants calibrate their bets in response to the ratio of box colors. RA was the primary measure for decision-making performance in this study considering heroin users were reported to have deficit in RA (11). A high RA score indicated a tendency to bet a larger proportion of available points on the high ratio rather than on the low ratio trials, whereas, a low RA score suggested the failure to use the available information in decision-making.

### Magnetic Resonance Experiments

The experiments were performed on a 3T MRI scanner (MR750, GE Healthcare, Milwaukee, WI, USA) with an 8-channel head radio frequency (RF) receive array coil. For anatomical image acquisition, a T2-weighted fast spin-echo sequence was employed with repetition time of 5,806 ms, echo time of 102 ms, number of averages of 1, 20 slices, slice thickness of 5 mm, and in-plane resolution of 0.45 mm × 0.45 mm. For rs-fMRI, functional images were acquired using a gradient-echo echo-planar imaging sequence with repetion time/echo time (TR/TE) of 2,000/30 ms, 40 slices, slice thickness of 3 mm, field of view of 230 mm, matrix size of 64 × 64, in-plane resolution of 3.6 mm × 3.6 mm, and 320 repetitions. For the purpose of feasibility, the MR experiments were arranged with months of lag after neurocognitive functioning assessments.

### Identification of Default Mode Network Subregions

**Figure 1:**
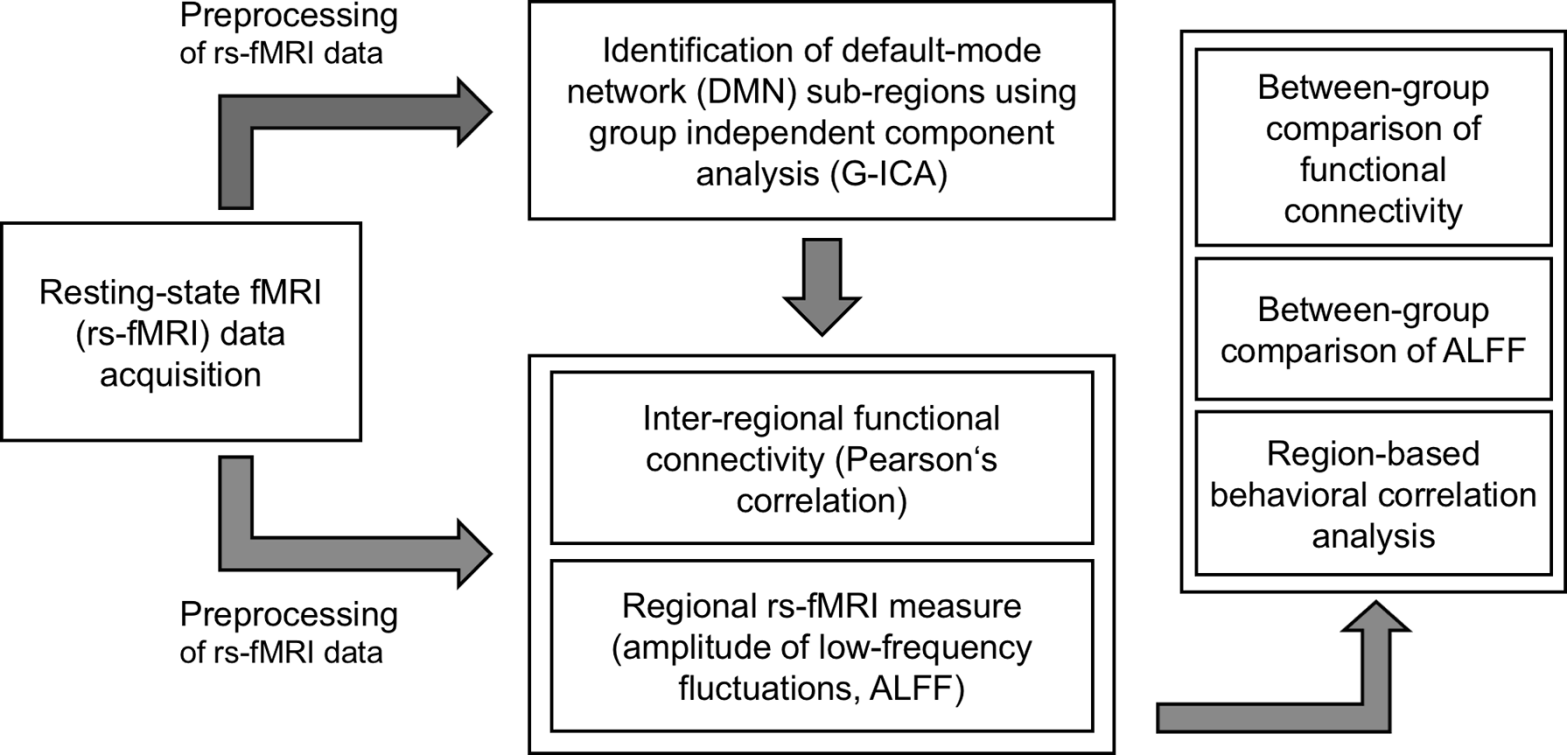
Overall analysis workflow of this study. The preprocessed resting-state functional magnetic resonance imaging (rs-fMRI) data was first used to identify the default mode network (DMN) subregions using group independent component analysis (G-ICA). The interregional functional connectivity was quantified by Pearson’s correlation, and the regional rs-fMRI measure [amplitude of low-frequency fluctuation (ALFF)] was also derived. The between-group difference of interregional functional connectivity and regional ALFF was assessed through a two-sample *t*-test with multiple comparisons. A region-based behavioral correlation analysis was performed through a model fitting approach controlling for group effect and multiple comparisons.

**Figure 2:**
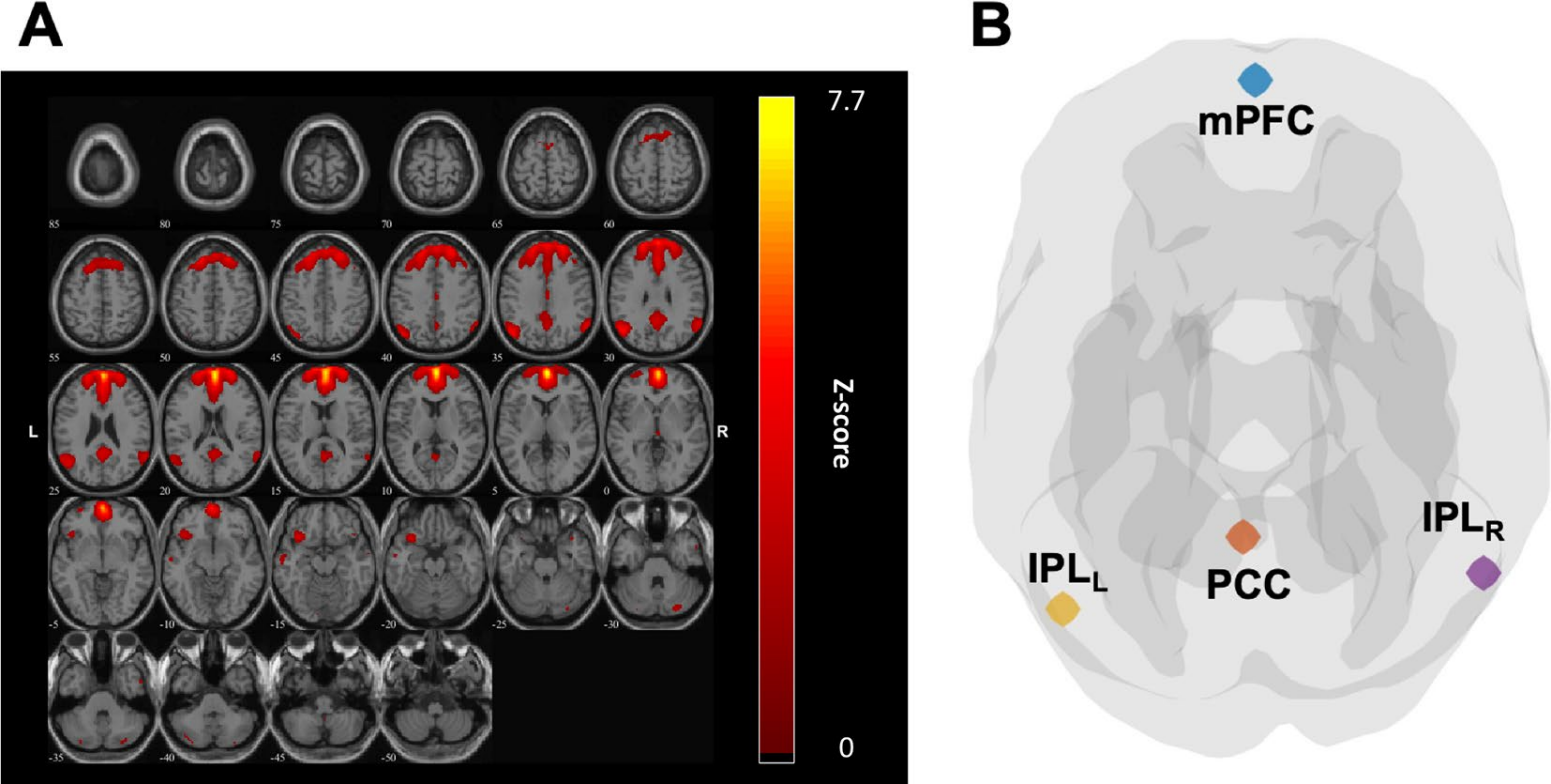
**(A)** The independent component (IC), which matched the default mode network (DMN), was used to extract the region of interest (ROI) of DMN subregions. **(B)** Four ROIs were identified from the IC as illustrated in panel **(A)** and clustered, including medial prefrontal cortex (mPFC), left inferior parietal lobe (IPL_L_), left inferior parietal lobe (IPL_R_), and posterior cingulate cortex (PCC).

The overall analysis workflow of this study is illustrated in **Figure 1**. To identify DMN subregions from the rs-fMRI data, we applied the group independent component analysis (G-ICA) approach on the rs-fMRI data of all participants from both groups (22 individuals). For each participant, the rs-fMRI data were first preprocessed using the Data Processing Assistant for Resting-State fMRI (DPARSF) toolbox (22) and Statistical Parametric Mapping (SPM) software program (https://www.fil.ion.ucl.ac.uk/spm/). The preprocessing procedures included realignment, slice timing adjustment using windowed Fourier interpolation, coregistration between fMRI and T2-weighted images, spatial normalization of T2-weighted images to Montreal neurological institute (MNI) space [International consortium for brain mapping (ICBM) 152 template] using Diffeomorphic anatomical registration through exponentiated lie algebra (DARTEL) (23), and spatial smoothing using Gaussian kernel with full width at half maximum of 5 mm. The preprocessing procedures for DMN identification differed from those for deriving functional connectivity and rs-fMRI measures, which are introduced in a subsequent section. G-ICA was performed on the preprocessed rs-fMRI data using the group ICA of fMRI toolbox (http://mialab.mrn.org/software/gift/). For each participant, the independent components (ICs) were extracted using the infomax ICA algorithm (24). To increase the reliability, we employed the ICASSO (25) algorithm, which could output the most reliable set of ICs from 100 repeated ICA runs. To determine the optimum number of ICs, we performed the G-ICA using four IC numbers (i.e., 10-, 15-, 20-, and 30-component ICAs were performed). For each IC, we calculated the similarity between the IC and a DMN template obtained from a previous study (26). The similarity of each IC was defined as the correlation coefficient (*r*) between its *Z*-score map and the *Z*-score map of the DMN template. For G-ICA results derived from each IC numbers (i.e., 10, 15, 20, and 30), the IC with highest similarity to the DMN template was selected as the matched component to represent the DMN. We then compared the four ICA settings and discovered that the matched IC obtained from 15-component ICA had the highest similarity (*r* = 0.559) to the DMN template. Therefore, this matched IC was identified and used to extract the DMN subregions (**Figure 2A**). The DMN subregions extraction was mainly based on the local maxima of the *Z*-score map of the matched IC. The matched ICs were divided into spatially connected clusters, and clusters containing less than 20 voxels were regarded as spurious clusters and were removed to avoid potential confounds. For each of the remaining cluster, the local maximum was identified as the center of the region-of-interest (ROI). The ROI was then extracted as a spherical region with a radius of 6 mm centering at the local maxima. Through this approach, in total, four ROIs within the DMN located at the mPFC, left inferior parietal lobe (IPL_L_), right inferior parietal lobe (IPL_R_), and PCC were identified and used for further analysis (**Figure 2B**).

### Interregional Functional Connectivity and Regional Resting-State Functional Magnetic Resonance Imaging Measure

To derive both the interregional functional connectivity and regional rs-fMRI measure, a dissimilar preprocessing approach from the one we used in DMN identification was employed on the rs-fMRI data of each participant. The preprocessing procedures included realignment, slice timing adjustment using windowed Fourier interpolation, coregistration between fMRI and T2-weighted images, spatial normalization of T2-weighted images to MNI space (ICBM512 template) using DARTEL (23), 24-parameter head motion correction (27), and nuisance covariate regression (white matter signals and cerebrospinal fluid signals). The rs-fMRI time series of each voxel were further detrended and band-pass filtered (0.01–0.1 Hz). We evaluated the maximum head motion for each participant and confirmed that it was within 3 mm (voxel size) among all participants. In addition, we performed a statistical comparison and confirmed that the two participant groups did not differ significantly in the amount of head motion (*p* = 0.931). On completing rs-fMRI data preprocessing, the mean time series of each ROI were obtained by averaging the voxel-wise preprocessed rs-fMRI time series within the selected ROI. To calculate the interregional functional connectivity, we first computed a Pearson’s correlation coefficient (PC) for connectivity measures in the time courses. Because the sample size is relatively small, we then adapted a Fisher’s *Z*-transformation for the PCs to stabilize the variance of correlations. In this study, we decided to use ALFFs to investigate the regional rs-fMRI activity within each ROI. ALFFs were derived from the voxel-wise preprocessed rs-fMRI time series (28) and accounted for the ALFFs of the rs-fMRI signals, in which the resting-state activity is considered to be more prominent.

### Statistical Analysis

The statistical analysis was composed of three parts as follows: functional connectivity analysis in the DMN, between-group comparison for the quantitative rs-fMRI measure (ALFF), and multiple linear model analysis for the association of the decision-making index (RA) and rs-fMRI measure (ALFF). To study the functional connectivity, the Fisher’s *Z*-transformation for the PCs was used to measure the significance of the difference between the two groups. In the study of functional connectivity, we had six sets of comparisons from four ROIs in the null hypothesis. To address the multiple comparison issue, a Benjamini–Hochberg (BH) procedure (29) was used to control a false discovery rate (FDR) at level 0.05. Specifically, for the *i*-th participant in the *j*-th group, *i* = 1,…,11, *j* = 1,2, the sample PC ${\hat{\rho }}_{j,k,k^{\prime },i}$ between the *k*-th and *k*’-th ROIs was computed from a set of pairs [(*Y* *_j,k,t,i_* ,*Y* *_j,k′,t,i_*): *t* = 1,…,320], where *Y* *_i,k,t,i_* and *Y* *_i,k’,t,i_* represent the mean rs-fMRI signals at time *t* across the *k*-th and *k*’-th ROIs, respectively, for the *i*-th participant in the *j*-th group. The sample correlation ${\hat{\rho }}_{j,k,k^{\prime },i}$ was then transformed to $Z_{j,k,k^{\prime },i}=0.5\ln\left(\frac{1+{\hat{\rho }}_{j,k,k^{\prime },i}}{1-{\hat{\rho }}_{j,k,k^{\prime },i}}\right)$ by a Fisher’s transformation. When the number of temporal points *T* is large, ${\bar{Z}}_{j,k,k^{\prime },i}$ approximately follows a normal distribution with mean $0.5\ln\left(\frac{1+\rho }{1-\rho }\right)$ and variance $\frac{1}{T-3}$ (30). In this study, the variance of *Z* *_j,k,k′,i_* is approximately 0.0032. ${\bar{Z}}_{j,k,k^{\prime }}=\sum_{i=1}^{11}Z_{j,k,k^{\prime },i}/11$ denotes an average of the transformed correlations between the *k*-th and *k*’-th ROIs in the *j*-th group. The transformed PCs have been standardized by corresponding variances in the Fisher’s *Z*-transformation. Therefore, with the Fisher’s transformation, the standard deviation for ${\bar{Z}}_{j,k,k^{\prime }}$ was 0.017 in all cases. *D* *_k,k’_* = *Z* *_1,k,k_* – *Z* *_2,k,k′_* denotes a difference in averages for the transformed PCs between the two groups. Considering the variance was estimated from the data, a two-sided *t*-test with degrees of freedom equal to 20 was used to evaluate the test statistic ζ*_k,k’_* = *D* *_k,k’_*/SD(*D* *_k,k’_*), where SD(*D* *_k,k’_*) denotes a standard deviation of *D* *_k,k’_*. This approach was then applied to test the significance of the functional connectivity difference between the MT and TC groups.

For the regional rs-fMRI activity, a two-sample *t*-test was used to evaluate the between-group difference of ALFFs. The BH procedure was again applied to control the FDR at level 0.05 for four sets of comparisons (corresponding to four ROIs).

The associations of the decision-making measure (RA) with fMRI signals were examined. *W* *_i_* denotes the values of RA for the *i*-th participant. Moreover, *X* *_i,k_* denotes the averaged ALFFs over the time period and across the *k*-th ROI for the *i*-th participant. δ*_i_* denotes an indicator variable for the group effect as δ_i_ = 1 if the *i*-th participant belongs to the MT group. We then fit a model by *W* *_i_* = *b*_0_ + *b*_1_ *X* *_i,k_* + *b*_2_δ*_i_*, *k* = 1,…,4. A two-sided *t*-test for each estimated coefficient was used to examine whether the coefficient was significantly away from zero. Given that we have to compare the ALFF from four ROIs, the BH procedure was again applied to control the FDR at level 0.05 for four sets of comparisons.

## Results

### Clinical Characteristics and Cambridge Gambling Task Measure (Risk Adjustment)

**Table 1** summarizes the clinical characteristics and CGT decision-making measures (RA) of MT and TC participants. The age of the participants in the MT group was 39.1 ± 5.9 years, which was significantly older than the age of the participants in the TC group (34.3 ± 4.1 years) (*p* < 0.01). At the rs-fMRI session, 54.5% (6/11) and 9.1% (1/11) of the participants in the MT group were tested positive for morphine and amphetamine, respectively. No use of these illicit drugs was detected in the participants in the TC group by urine testing. The CGT measure (i.e., RA) was not significantly different between the MT and TC groups.

**Table 1:** Clinical characteristics and Cambridge gambling task (CGT) measure [risk adjustment (RA)] of heroin users on methadone treatment (MT) and therapeutic community (TC) program.

	MT (*n* = 11)		TC (*n* = 11)		t or x^2^	p
	M/*n*	SD/%	M/*n*	SD/%		
**Age, years**	39.14	5.93	34.28	4.09	−2.46	<10^−2^
**Education, years**	8.55	1.75	9.82	1.40	0.15	0.17
**Handedness Preference Index** **^1^**	81.82	54.88	74.24	44.44	0.35	0.73
**Religiosity**						
Chinese folk religions^2^	11	100.0	0	0		
Christian	0	0	11	100.0		
**Heroin onset age, years**	23.27	4.84	19.36	4.37	−1.99	0.10
**Methadone daily dose, mg**	54.47	20.82				
**Length of treatment, months**	12.00	10.12	43.18	19.67	4.68	<10^−3^
**Current psychiatric disorder**						
Any affective disorders	1	9.09	0	0	1.05	0.31
Any anxiety disorders	0	0	2	18.18	2.20	0.14
**Urine drug testing**						
Morphine +	6	54.54	0	0	8.25	<10^−2^
Amphetamine +	1	9.09	0	0	1.05	0.31
**Days of drug use in past 4 weeks**						
Alcohol, days	3.18	5.17	0	0	−2.04	0.10
Heroin, days	9.64	10.87	0	0	−2.94	<10^−2^
Methamphetamine, days	0.36	0.67	0	0	−1.79	0.10
Cambridge gambling task*						
Risk adjustment (RA)	1.06	1.31	1.26	1.02	0.63	0.56

*For TC, only 10 participants completed the CGT.

^1^Handedness Preference Index was measured using Briggs–Nebes modified Annett’s Hand Preference Questionnaire.

^2^Chinese folk religions include Buddhism and Taoism.

### Identification of Default Mode Network Subregions By Independent Component Analysis

As illustrated in **Figure 2A**, the subregions of DMN for each group were identified using the ICA approach. The most closely corresponding components exhibiting DMN characteristics were selected for the following connectivity and statistical analysis (**Figure 2B**).

### Between-Group Comparison of Functional Connectivity

**Figure 3:**
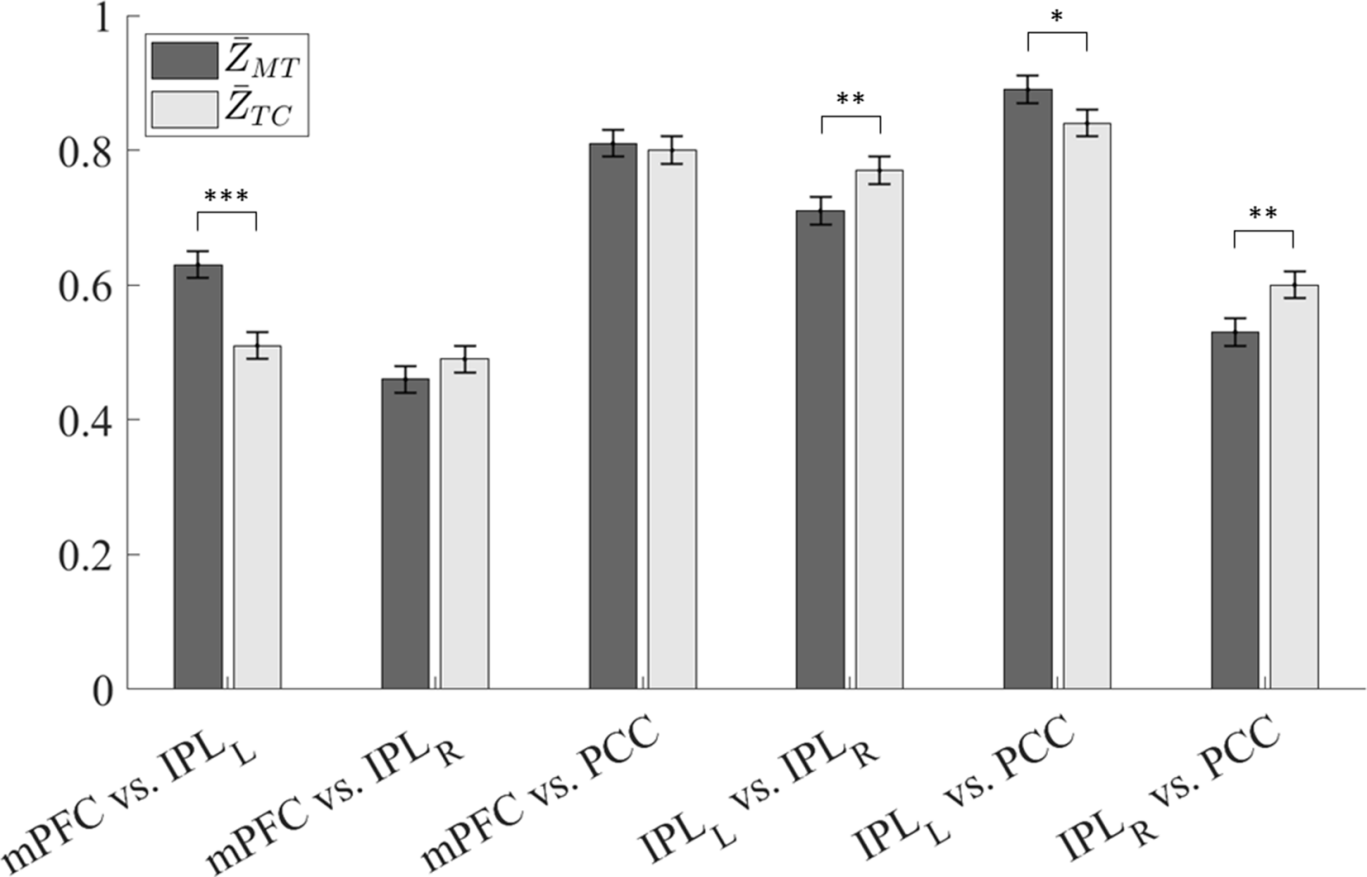
Interregional resting-state functional magnetic resonance imaging (rs-fMRI) functional connectivity of the MT and TC groups. The functional connectivity was presented as the Fisher’s *Z*-transformation of the Pearson’s correlation (*p < 0.05; **p < 0.01; ***p < 0.001).

The results of functional connectivity analysis are presented in **Table 2** and **Figure 3**. Herein, the subregions 1 to 4 represented the mPFC, IPL_L_, IPL_R_, and PCC subregions in DMN, respectively. For the MT and TC groups, the averages of the transformed PCs between two subregions, *k* and *k*’, were denoted by ${\bar{Z}}_{MT,k,k^{\prime }}$ and ${\bar{Z}}_{TC,k,k^{\prime }}$, respectively. The values of ${\bar{Z}}_{MT,1,2}$, ${\bar{Z}}_{MT,1,3}$, ${\bar{Z}}_{MT,1,4}$, ${\bar{Z}}_{MT,2,3}$, ${\bar{Z}}_{MT,2,4}$, and ${\bar{Z}}_{MT,3,4}$ were 0.63, 0.46, 0.81, 0.70, 0.89, and 0.53, respectively, and the values of ${\bar{Z}}_{TC,1,2}$, ${\bar{Z}}_{TC,1,3}$, ${\bar{Z}}_{TC,1,4}$, ${\bar{Z}}_{TC,2,3}$, ${\bar{Z}}_{TC,2,4}$, and ${\bar{Z}}_{TC,3,4}$ were 0.51, 0.49, 0.80, 0.77, 0.84, and 0.60, respectively. The values of the difference between groups D_1,2_, D_1,3_, D_1,4_, D_2,3_, D_2,4_, and D_3,4_ were 0.12 (*p* < 0.001), −0.03 (*p* = 0.23), 0.01 (*p* = 0.68), −0.07 (*p* = 0.0085), 0.05 (*p* = 0.050), and −0.07 (*p* = 0.0085), respectively, with the standard deviation at a value of 0.024 for all cases.

**Table 2:** Analysis results of the difference of functional connectivity between the MT and TC groups. The tests were based on Fisher’s *Z*-transformation for the Pearson’s correlations.

	${\bar{Z}}_{\text{MT}}$	${\bar{Z}}_{\text{TC}}$	Difference	SD	*p*-value
mPFC vs. IPL_L_	0.63	0.51	0.12	0.02	<0.001
mPFC vs. IPL_R_	0.46	0.49	−0.03	0.02	0.23
mPFC vs. PCC	0.81	0.80	0.01	0.02	0.68
IPL_L_ vs. IPL_R_	0.71	0.77	−0.07	0.02	0.0085
IPL_L_ vs. PCC	0.89	0.84	0.05	0.02	0.050
IPL_R_ vs. PCC	0.53	0.60	−0.07	0.02	0.0085

mPFC, medial prefrontal cortex; IPL_L_, left inferior parietal lobe; IPL_R_, right inferior parietal lobe; PCC, posterior cingulate cortex.

Using the BH procedure to control the FDR at level 0.05 results in thresholds for *p*-values in a descending order as 0.008, 0.017, 0.025, 0.033, 0.042, and 0.05. Therefore, after conducting the BH procedure to screen out *p*-values, we concluded that D_1,2_ (*t*-ratio = 5.0), D_2,3_ (*t*-ratio = 2.91), and D_3,4_ (*t*-ratio = 2.91) were significantly away from zero.

### Between-Group Comparison of Regional Resting-State Functional Magnetic Resonance Imaging Measure (Amplitude of Low-Frequency Fluctuation)

The two-sample *t*-test conducted with the BH procedure to control the FDR revealed there was no significant difference among all cases.

### ROI-Based Behavioral Association

**Figure 4:**
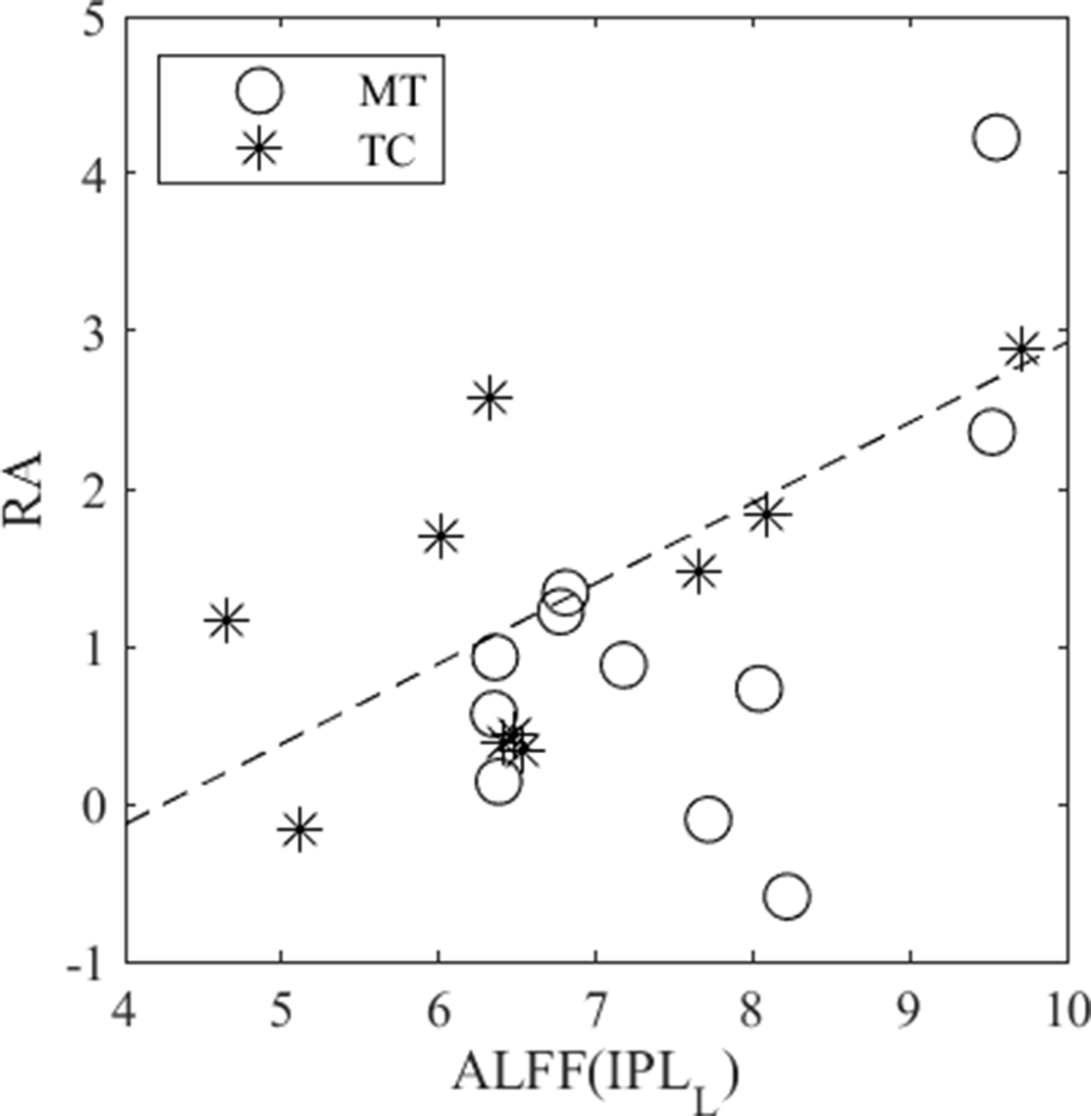
A significant correlation (b1 = 0.51, *t* = 3.005, *p* = 0.007) was revealed between RA and ALFF in IPL_L_ when controlling for group effect. The MT and TC groups were denoted by circles and stars, respectively.

The estimation results for the association between ROI-based rs-fMRI measure (ALFF) and CGT measure (RA) are presented in **Table 3** and **Figure 4**. Our results revealed that, after conducting the BH procedure to control the FDR, RA exhibited a significant association with ALFFs in IPL_L_ (_1_ = 0.51, t = 3.005, *p* = 0.007).

**Table 3:** Estimation results for the association between RA and ALFF in IPL_L_ with an adjustment for group effect.

	Estimate	SD	*t* value	*p*-value
Intercept	−2.17	1.19	−1.83	0.08
ALFF(LIP_L_)	0.51	0.17	3.005	0.007
Group	−0.63	0.46	−1.38	0.18

## Discussion

The present study investigated the rs-fMRI functional connectivity of DMN and its correlation with decision-making performance in heroin users on MT and medication-free TC program. More than half of the participants in the first group were tested positive for illicit opioids use, whereas, those in the second group were abstinent from any illicit drugs for an average of 5 years. To the best of our knowledge, this study was one of the few to investigate the brain functioning and its behavioral correlates for heroin users with diverse treatment modalities and outcomes. The study revealed that TC had increased rs-fMRI functional connectivity in IPL_L_–IPL_R_ and IPL_R_–PCC and decreased rs-fMRI functional connectivity in mPFC–IPL_L_, compared with MT. These findings suggested the potential effects of treatment on DMN functional connectivity among heroin users.

Prior studies have reported that heroin users have altered functional connectivity in DMN compared with healthy controls (14–16, 31, 32). The reduced functional connectivity might be related to the relapse of heroin use (17). The present study discovered that IPL might play a key role in the various patterns of DMN connectivity between MT and TC. Earlier studies have implicated the involvement of IPL in the treatment of addictive disorders. In comparison with healthy controls, the abstinent former but not current cocaine users had overactivated IPL_R_ (33). The cocaine-dependent patients who were more engaged in cognitive-behavioral therapy exhibited a greater reduction of task-related neural activity in IPL (34). Recently, a study revealed that heroin users on MT had overactivated IPL_R_ in cue-reactivity tasks, in comparison with those individuals on protracted abstinence (35). Therefore, the potential role of IPL as a neural target of therapeutic interventions for addictive disorders warrants further investigation.

The analysis of neural correlates for RA, which quantifies bet calibration across trials with various ratios of favorable outcomes in CGT, revealed that it was positively associated with ALFF in IPL_L_ for all participants when controlling for group effect. The IPL has been implicated across a wide array of cortical function domains (36, 37). Specifically, the IPL was involved in the process of valuation of various choices, numerical representation, and information integration, which were closely related to reward-based decision-making. Previous studies have demonstrated that the IPL activity increased in uncertain reward-based decision-making (38, 39). Results from a meta-analysis also revealed that IPL was more activated in the anticipation phase than in the outcome phase during decision-making (40). Our findings supported the association of IPL functioning with the encoding probability of uncertain outcomes and the calculation of utility in risky decision-making. Notably, the link between the functioning of DMN components and CGT measures was not modified by treatment. This warrants further investigation on the treatment effects on the underpinning neural networks related to decision-making.

Several limitations should be addressed in this study. First, the number of participants per group was relatively small. In addition, both groups differed in religiosity, comorbid drug, and mental problems, which might have confounded the associations with treatment modalities and outcomes. However, the adjustment for covariates was hampered by the relatively small sample size. Given that Operation Dawn provides the largest faith-based residential program in Taiwan, there are dozens of new participants annually (41). Including more collaborative organizations may improve the efficiency of participant enrollment and increase the sample size; however, the tradeoff of heterogeneous programs should be considered (42). Second, the causality relation between treatment and brain functional connectivity could not be established in this cross-sectional study. Because rs-fMRI functional connectivity was potentially a trait marker (43), the treatment effects should be examined with a follow-up design. Third, the MR experiments and CGT were performed separately. The confounding effects of time-dependent events, such as drug exposure, might bias the associations between brain functioning and behavioral measures. Finally, only the intraregional and interregional DMN functional connectivity were investigated in this study. This warrants further investigation of other networks potentially involved in heroin addiction, such as salience network and executive control network. Moreover, state-of-the-art approaches with graph theoretical analysis should be used in future studies (43).

## Conclusion

In summary, our study demonstrated that the rs-fMRI technique could be used to delineate the altered intraregional connectivity in DMN for heroin users with various treatment regimens and diverse outcomes. This is one of the few studies to enroll medication-free, abstinent heroin users to investigate their brain functioning in the long-term recovery process. Our results indicated the significant difference of rs-fMRI connectivity between the two treatment groups in numerous connections between IPL and other DMN subregions, including IPL_L_–IPL_R_, IPL_R_–PCC, mPFC–IPL_L_, and IPL_L_–PCC. In addition, the regional rs-fMRI metric, ALFF, demonstrated a significant association with RA, specifically in the IPL_L_ subregion, which is consistent with previous reports. Our findings prompted us to further verify that the use of neuroimaging techniques could facilitate the understanding of the relationship between brain functioning and heroin use, as well as the effects of various treatment modalities.

## Ethics Statement

This study was carried out in accordance with the recommendations of Institutional Review Board, National Health Research Institutes, with written informed consent from all subjects. All subjects gave written informed consent in accordance with the Declaration of Helsinki. The protocol was approved by the research ethics committees of National Health Research Institutes (IRB#: EC0990801).

## Author Contributions

SW and HL designed the study, and conducted the patient recruitment and experimental data acquisition. LK, PL, SL, ML, HJ, and SW analyzed and interpreted the data. LK, PL, SL, ML, HJ, and SW wrote the manuscript. All authors have reviewed the manuscript and approved the submission.

## Funding

This work was supported in part by the National Health Research Institutes (LK: BN-108-PP-06; SW: NP-103-PP-05 and NP-103-SP-04), Ministry of Science and Technology (LK: 106-2420-H-400-001-MY2 and 107-2221-E-400-001; HL: 98-2314-B-038-026-MY2), and the Central Government Science and Technology grant (LK: 107-1901-01-19-02 and 108-1901-01-19-08).

## Conflict of Interest Statement

The authors declare that the research was conducted in the absence of any commercial or financial relationships that could be construed as a potential conflict of interest.
